# Group Membership, Group Change, and Intergroup Attitudes: A Recategorization Model Based on Cognitive Consistency Principles

**DOI:** 10.3389/fpsyg.2018.00479

**Published:** 2018-04-06

**Authors:** Jenny Roth, Melanie C. Steffens, Vivian L. Vignoles

**Affiliations:** ^1^Department of Psychology, Social Psychology, University of Würzburg, Würzburg, Germany; ^2^Faculty of Psychology, Social, Environmental, and Economic Psychology, University of Koblenz-Landau, Landau, Germany; ^3^School of Psychology, University of Sussex, Brighton, United Kingdom

**Keywords:** group change, recategorization, intergroup bias, prejudice, social identification, cognitive dissonance, cognitive balance, identity integration

## Abstract

The present article introduces a model based on cognitive consistency principles to predict how new identities become integrated into the self-concept, with consequences for intergroup attitudes. The model specifies four concepts (self-concept, stereotypes, identification, and group compatibility) as associative connections. The model builds on two cognitive principles, balance–congruity and imbalance–dissonance, to predict identification with social groups that people currently belong to, belonged to in the past, or newly belong to. More precisely, the model suggests that the relative strength of self-group associations (i.e., identification) depends in part on the (in)compatibility of the different social groups. Combining insights into cognitive representation of knowledge, intergroup bias, and explicit/implicit attitude change, we further derive predictions for intergroup attitudes. We suggest that intergroup attitudes alter depending on the relative associative strength between the social groups and the self, which in turn is determined by the (in)compatibility between social groups. This model unifies existing models on the integration of social identities into the self-concept by suggesting that basic cognitive mechanisms play an important role in facilitating or hindering identity integration and thus contribute to reducing or increasing intergroup bias.

## Introduction

People belong to social groups. Being a member of one social group (i.e., in-group) often implies not to be a member of another social group (i.e., out-group). In-groups – in contrast to out-groups – form part of the individual’s self-concept. They constitute people’s social identity (Tajfel, 1974). The distinction between in-group and out-group fosters intergroup bias and prejudice (Allport, 1954; Brewer, 1979; Otten and Wentura, 1999; Ashburn-Nardo et al., 2001; Hewstone et al., 2002). In most of the contemporary literature on group membership and intergroup bias, group membership is treated as a given fact that can be more or less salient depending on contextual factors (e.g., Brewer and Weber, 1994; Ensari and Miller, 2001; Gaertner and Dovidio, 2005; Jones and Jetten, 2011). By implication, a given group membership should affect psychological processes and behavior in some situations but less so in others. However, beyond such short-term contextual fluctuations in identity salience, people also undergo long-term change in some of their group memberships. For example, people change working teams, residence, or political party affiliation. They become a non-smoker, age, or gain weight. Increasingly, people are also geographically mobile—as reflected in the current migration stream to European countries but also in migration between and within counties and states all over the world (International Organization for Migration, 2018). These changes confront people with new identities. One of the major challenges that goes along with migration and other changes in group membership is that people successfully integrate new identities into their self-concept. If integration of norms, values, and attitudes of new and old identities fails, changes in group membership may end in intergroup hostility and discrimination jeopardizing the functioning of a society. Therefore, it is important to investigate the psychological mechanisms involved in changes in group membership.

Previous research into social identity change has focused most strongly on short-term fluctuations in identity salience, but the examples above highlight that some changes in group identities are likely to be more enduring. Whereas the term “identity salience” usually refers to recategorizing the self-depending on temporary contextual fluctuations, with the term “group change” in the present paper, we refer to recategorizing the self independently from temporary contextual fluctuations. We use group change as an umbrella term that refers to changes in group membership from an in-group to a previous out-group in people’s course of life. These changes in group membership have various reasons and differ on various dimensions. Considering the large body of research on intergroup attitudes and the strong interest in changing intergroup bias to prevent intergroup hostility and discrimination, it is remarkable that there is little insight into how changing group membership, such that one leaves one’s in-group and becomes a member of a former out-group, affects intergroup attitudes. Even though there is research on the effectiveness of recategorization and cross-categorization in reducing intergroup bias, this research focuses on the inclusiveness of salient in-groups but not on a change in the individual’s standing as a member of one group or the other (Brewer, 2000; Gaertner et al., 2000; Dovidio et al., 2006). With the present article we aim to shed light on how changes in the individual’s standing as a member of a previous and a new in-group affects the integration of these groups into the self-concept with consequences for intergroup attitudes. We present a model focusing on the cognitive mechanisms that may help or hinder the integration of new identities into the self-concept. Research on these mechanisms can inform policy to derive strategies that will help identity integration—and thus social cohesion—in the face of this pressing societal concern.

Previous models provide valuable insight into distinct stages of identity integration (Amiot et al., 2007) or types of identity integration (e.g., Roccas and Brewer, 2002; Huynh et al., 2011). An acculturation perspective has mostly inspired these models. All of these models mention that the overlap or match between cultural groups affects identity integration processes. However, they are less explicit about how the match and mismatch between cultures is represented in the cognitive knowledge structure with consequences for identification and intergroup attitudes. The present recategorization model unifies previous models by suggesting that the integration of social groups into the self-concept follows cognitive consistency principles. More precisely, the present model suggests that the integration of social groups into the self-concept depends on the (in)compatibility of a new group membership with previous memberships and that the more a certain group is integrated into the self-concept the more this group is preferred to those that are less integrated into the self-concept. Thus, the present model suggests a unifying and general mechanism underlying the integration of previous and new social groups into the self-concept with consequences for implicit and explicit intergroup attitudes; the model can be applied to cultural groups as well as all other kinds of social groups. The sources of intergroup attitudes are, of course, multiple and complex. They include macro-level cultural and historical processes, interpersonal and intergroup interaction processes, as well as motivational dynamics. In the present model, we focus on basic cognitive processes that contribute to social identity and to intergroup attitudes. Importantly, we do not claim that the mechanism we focus on here is the most relevant or the sole. However, we believe that it forms an important part of understanding the integration of social identities into the self-concept, intergroup attitudes, and the change of intergroup attitudes when people change group membership.

In the following, we first review a model by Greenwald et al. (2002) that provides the theoretical base for the present recategorization model. Then, we present a crucial extension of Greenwald et al.’s (2002) model to predict when people integrate new social groups into their self-concept and outline hypotheses derived from the recategorization model. Next, we review literature demonstrating that group membership contributes to implicit and explicit intergroup attitudes. We present an inductive mechanism explaining categorization-induced intergroup bias because of mentally associating the self-concept with an in-group. Based on the model, we propose that explicit as well as implicit intergroup attitudes can be affected by changing group membership and that this effect depends on mentally associating groups to the knowledge structure of the self. We suggest that this process depends on cognitive consistency principles. We argue that this recategorization model offers an integrative and dynamic understanding of how changing group membership affects the integration of new groups into the self-concept and consequently intergroup attitudes. Thereby, we connect the literature on explicit and implicit attitude change with the literature on intergroup relations and unify basic assumptions of previous models on identity integration. Predictions derived from the recategorization model can inspire systematic research on an often neglected but highly relevant issue in contemporary societies.

## Overview of Theory

The present recategorization model builds on the unified theory of implicit attitudes, stereotypes, self-concept, and self-esteem (Greenwald et al., 2002). Its premise is that social knowledge is represented in associative connections between concepts and attributes and that the formation of cognitive associations follows cognitive consistency principles. Greenwald et al. (2002) suggest a balanced identity design in that information about the self, social groups, and their attributes as well as associated valence is stored in a cognitively consistent manner and that the cognitive system resists forming associations that result in cognitive dissonance. In the present model, we use the terms as specified in Greenwald et al. (2002). Therefore, we will review the elements of their model that are relevant to the present model before we present an extension of it to account for the integration of social groups into the self-concept, from which we derive predictions for intergroup attitudes when people change group membership.

### Theoretical Foundation and Definitions

**FIGURE 1:**
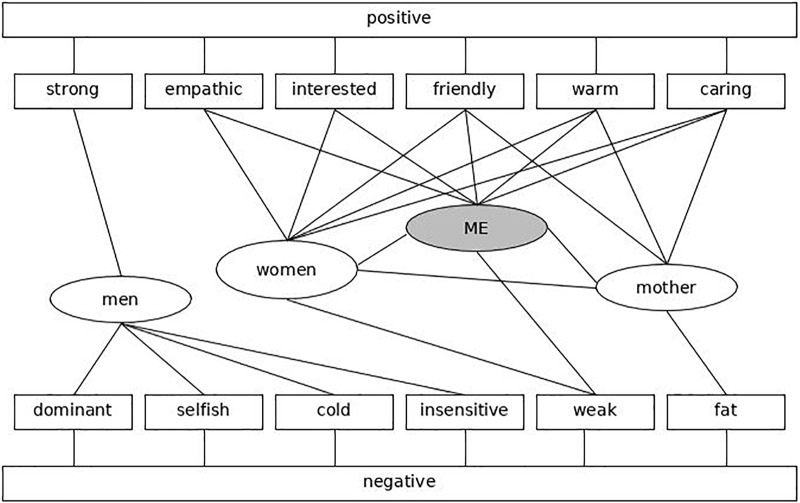
(Own visualization based on Greenwald et al., 2002) Presents an example of a very simplified associative knowledge structure that includes aspects that correspond to the psychological constructs self-concept, self-esteem, stereotypes, prejudice, and group identification. In this example, the social group women is most strongly associated with positive traits whereas the social group men is most strongly associated with negative traits. Thus, whereas in this simplified example the group women is represented predominantly positively, the group men is represented mostly negatively.

The concepts that are relevant for the present model are the self, groups, and attributes. These concepts are represented in the model as cognitive nodes (Greenwald et al., 2002) (**Figure 1** for a simplified illustration of a cognitive knowledge structure). The *Me*-node, for example, contains associations that organize information about personal experiences. Associative connections link different concepts. These associations vary in strength. The stronger the association, the higher the probability that cuing one concept will result in the activation of the associated concept (Hebb, 1949). Along these lines, “self-concept” is the term used to represent associations of the *Me*-node with traits. “Self-esteem” is closely related to the self-concept as the former represents the overall valence of the traits that are associated with the *Me*-node. “Group identification” is represented in the associations between the *Me*-node and a group-node. As for all associative connections presented here, group identification can vary in strength between individuals who belong to the same social groups. “Intergroup attitudes” refer to the relative evaluation of one social group (e.g., women) compared to another social group (e.g., men). “Evaluative intergroup bias” mirrors the preference for one social group over another social group. Bias is grounded in stronger associations of one social group with positive traits and less negative traits compared to another social group.

### Basic Propositions and Principles

In line with Greenwald et al. (2002), a major proposition of the present model is that most individuals show self-positivity. Their self is associated with more positive than negative traits and the resulting overall valence is positive. This proposition is based on the vast empirical evidence that shows that most people evaluate themselves in more positive ways than others (e.g., Bosson et al., 2000; Greenwald and Farnham, 2000; Franck et al., 2007; Olson et al., 2007; Yamaguchi et al., 2007; Roth et al., 2012; but see Falk and Heine, 2015, for cross-cultural differences in implicit self-esteem). Another empirical observation that underscores this proposition is that most people perceive themselves better than average on dimensions that they value (Alicke and Govorun, 2005; Brown, 2012).

**FIGURE 2:**
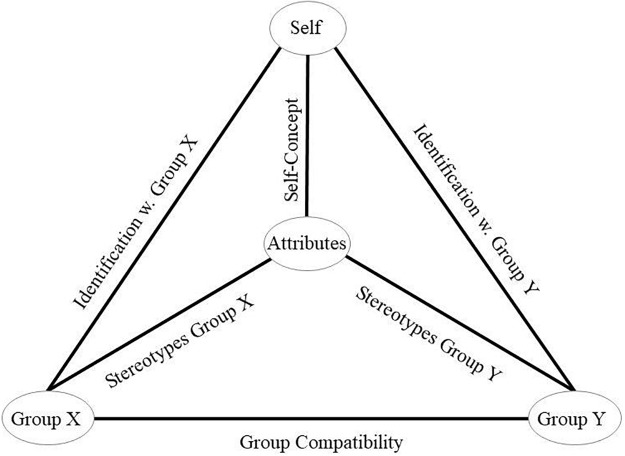
A simplified model of identity integration. Each oval represents a concept. Lines between concepts represent associations. The left and the right upper triangle correspond to a balanced identity design as represented in Greenwald et al. (2002). If a person identifies with Group X, and the self is associated with certain attributes the balance congruity and the dissonance imbalance principle suggest that Group X is also associated with the same attributes. Similarly, when the self is associated with certain attributes and so is Group X then, identification with this group strengthens. Finally, the self-concept may also be influenced by strong identification with a Group X that is associated with certain attributes. For example, the stereotypes that a person associates with a social group may be associated with the self the more a person identifies with that social group. Similarly, the traits that a person associates with the self may be associated with the group the more the person identifies with the group. Finally, when the same traits are ascribed to the group and to the self the association between the self and the group may strengthen. The same holds true for the right upper triangle if Group X is replaced by Group Y. For a more detailed description, see text above. The lower triangle extends the design along cognitive congruency principles. The balance–congruity principle and the imbalance–dissonance principle predict that only if both groups Group X and Group Y are compatible, both groups may show strong associations with the self. If groups are incompatible with each other only either one of both groups will show stronger associations with the self, whereas the other group will show weaker associations with the self.

The present model builds on two principles that Greenwald et al. (2002) derived from Heider’s (1958) balance theory, Osgood and Tannenbaum’s (1955) congruity theory, and Festinger’s (1957) cognitive dissonance theory. The first principle, the balance–congruity principle, suggests that when two nodes in the associative network structure are simultaneously linked to another third node, the association between both nodes would strengthen. For example, if the *Me*-node is strongly associated with the group node *women* (i.e., the person identifies with women) and strongly associated with the trait *warm* (i.e., the self-concept includes the trait warm) the balance–congruity principle will strengthen the association between *women* and *warm* (i.e., the group stereotypes include the trait warm). Associative principles are supposed to work bi-directionally. Therefore, the balance–congruity principle also predicts that when the *Me*-node is associated with the group-node *women* and similarly *women* is associated with the attribute *warm*, the association between the *Me*-node and the attribute *warm* will strengthen. Similarly, when the *Me-*node is associated with the attribute *warm* and the group-node *women* is associated with the same trait, then the balance–congruity principle will strengthen the association between the *Me*-node and *women* (see **Figure 2**, upper left triangle if Group X is replaced by women, or if Group Y is replaced by women, the same example holds true for the right upper triangle). The second principle, the imbalance–dissonance principle, suggests that no associations will be formed between a node and a third aspect of the network when this would imply the association of two nodes that are diametrically opposed. This principle predicts that the neural network resists forming a strong association between the self and a social group when the group is associated with attributes that contradict the attributes that are associated with the self. Thus, groups that are strongly associated with the self should share the attributes associated with the self more than groups that are less strongly associated with the self. When the self is associated with positive traits more than with negative traits, then the balance–congruity principle and the imbalance–dissonance principle predict that groups that are associated with the self should be associated with more positive traits than groups that are not associated with the self (Greenwald et al., 2002).

Before we present a crucial extension of Greenwald et al. (2002), we shortly highlight connections to what researchers have suggested to be two distinct routes to group identification. Previous research has pointed out that group identification is established from two distinct routes: self-stereotyping and self-anchoring (van Veelen et al., 2015). The present model is in line with this literature since it is based on the proposition that associative links work in a bidirectional way. Within the present model, the balance–congruity principle suggests that the association between the self and a social group, thus group identification, strengthens when the self and the social group share associations with traits. Self-stereotyping describes the process that traits associated with the social group are also associated with the self. Self-anchoring describes the process that traits associated with the self are also associated with the social group. Thus, associations between the self, the social group, and traits strengthen to result in cognitive consistency between these aspects of the knowledge structure. The present model complements van Veelen et al.’s (2015) model on identification processes by adding that self-stereotyping and self-anchoring are processes leading to identification that can be understood as two mechanisms based on the balance–congruity principle.

### A Recategorization Model

Drawing on the extensive evidence that supports cognitive consistency principles in general and Greenwald et al.’s (2002) model specifically (Cvencek et al., 2012), we propose a model based on cognitive consistency processes that specifies the integration of new social groups into the self-concept with consequences for intergroup attitudes. The present recategorization model is a logical extension of Greenwald et al.’s (2002) previous model. **Figure 2** depicts a balanced identity design that includes the concepts self, attributes, and two group-concepts, Group X and Group Y. These concepts are illustrated as nodes. Lines that connect the nodes represent the social-cognitive constructs group identification, self-concept, and stereotypes. These lines represent associations between nodes. Crucial for the present model, as new construct, we implemented the (in)compatibility between social groups. This construct describes whether groups align in their stereotypes, norms, values, goals, etc. Two groups are said to be compatible when their stereotypes, norms, values, goals, etc., are in line with each other’s. They are incompatible when their group’s stereotypes, norms, values, goals, etc., contradict each other or when groups have conflicting goals or compete over resources. For example, the group vegetarians is incompatible with the group of carnivores, whereas the groups vegetarians and vegans are less incompatible (or more compatible).

#### Recategorization, Group-(In)Compatibility, and Stereotypes

The present recategorization model builds on the same basic propositions and principles as outlined above. When these principles are applied to an associative network that consists of more than one social group then, the balance–congruity principle suggests that a social group is associated with the self more strongly when the group is compatible with other social groups that already are associated with the self. Incompatibility in this respect means that one of the social groups shares associations to traits that are diametrically opposed to the traits that are shared with another social group. Incompatibility may also refer to groups that are defined as mutually exclusive such as vegetarian and carnivore or liberals and conservatives. The balance–congruity principle predicts that associations between two social groups form when both groups share the associations to the attributes. The imbalance–dissonance principle predicts that the network will resist forming associations between two social groups that are diametrically opposed, which means incompatible or mutually exclusive. Thus, a new social group will be associated more strongly with the self when it is compatible with another social group that already shows associations with the self. The network will refrain from forming associations with a social group that is incompatible with another social group that already is associated with the self (see lower triangle illustrated in **Figure 2**). As outlined above, the model suggests bi-directionality of associations. Therefore, the principles also suggest that if two social groups are both strongly associated with the self these groups are perceived to be more compatible with each other than two social groups that differ in the associative strength with the self. However, this reverse process is not essential for the key question of how new social groups become integrated into the self-concept. Therefore, in the following we will focus on how (in)compatibility of a previous and a new in-group affects group identification rather than how group identification affects perceived (in)compatibility between social groups.

#### Predictions for How Changing Social Groups Affects Group Identification

Based on the balance–congruity and the imbalance–dissonance principles, in the following, we derive predictions for associations of social groups with the self (i.e., group identification), resistance from associating social groups with the self, and a decrease in the associative strength between one social group with the self in favor of a stronger associative link with another social group and the self.

##### Incompatible social groups imply discrepancies in group identification

When people change from one social group to another social group and both groups are incompatible with each other, then the model’s balance–congruity and imbalance–dissonance principles predict that one of the two groups will be more strongly associated with the self than the other social group. There are two cognitive routes that lead to discrepancies in group identification. *Dissociating* the previous in-group from the self or *resisting* to form associations between the self and the new in-group. Dissociating the previous in-group from the self in favor of the new in-group implies a shift in group identification. Both processes lead to the same outcome: Changing groups that are incompatible with each other leads to stronger associations of one of the two social groups with the self than associations of the other social group with the self. Similarly, when the previous and the new in-groups are mutually exclusive, people will identify more strongly with one or the other group. This leads to the first prediction:

Hypothesis 1: Discrepancies in identification with a previous and a new in-group will be stronger to the extent that the two groups are incompatible.
The bi-directional nature of the model implies the prediction that when people strongly identify with one social group but disidentify with another social group these two groups are perceived as more incompatible than two social groups that people identify similarly strong with.

On the one hand, changes in group membership can be imposed by external pressure. For example, a person may be forced into unemployment and find himself or herself as a welfare recipient despite having strongly integrated an employee identity into the self-concept. Since a welfare recipient identity precludes an employee identity—both social groups are diametrically opposed and thus, incompatible with each other or mutually exclusive—people either associate the employee identity more strongly with their self or they associate the welfare recipient identity more strongly with their self. Which of both social groups is more strongly associated with the self depends on which of the social groups shares attributes that are associated with the self. Thus, if a person describes herself or himself as hardworking, independent, and successful, the cognitive network of this person resists forming strong associations with welfare recipients. On the other hand, a person may choose to become member of a new social group. Similarly, then, the associations between the social groups and the self are determined by the incompatibility between the new in-group and the previous in-group. For example, a person who was a carnivore and chooses to become a vegetarian associates carnivores or vegetarians more strongly with the self in order to maintain cognitive consistency. When people choose a new group membership, however, they may already associate the self with traits that are more strongly associated with the new in-group than with the former one. Then, the previous in-group is dissociated from the self in favor for the new in-group being associated with the self to maintain cognitive consistency.

Hypothesis 2: When a new in-group is incompatible with a previous in-group, identification with the new in-group will be stronger if people choose to become a member of the new in-group than if they were forced to become a member of the new in-group.

##### Compatible social groups imply expanding identities

When people change from one social group to another social group and both groups are compatible with each other or overlapping, then the model predicts that both groups are associated with the self to a similar extent. As was the case for incompatible identities, changes in group membership between compatible identities can be imposed. For example, a person may be forced into another employment despite having strongly integrated a previous employee identity into the self-concept. If both employee identities share central characteristics—both social groups are compatible with each other—people associate the new employee identity in accordance with the previous employee identity with the self. Similarly, when the previous and the new in-groups are highly overlapping, people may identify in similarly strong ways with both groups. Thus, if a person describes herself or himself as hardworking, independent, and successful, and these characteristics are associated with the previous as well as with the new employee identity, then the cognitive network expands, forming association with the new in-group in accordance with the previous in-group. If a person chooses to become a member of a new social group, similarly the associations between the social groups and the self are determined by the compatibility between the new in-group and the previous in-group. To draw on the above-mentioned example, a person who was a vegetarian and chooses to become vegan simultaneously associates vegetarians and vegans with the self when both are compatible.

Hypothesis 3: Compatibility of new in-groups with previous in-groups that people already show strong identification with will result in similarly strong identification with the new and the previous in-groups irrespective of whether the new in-groups are chosen or imposed.

Before we derive hypotheses for how changing group membership affects intergroup attitudes, we will summarize established findings on how group membership and group identification contribute to intergroup attitudes.

## Group Identification and Intergroup Attitudes

As stated above, people form stronger associations between the self and social groups that share the attributes that are associated with the self (also see Turner et al., 1987). The premise that most people have positive self-esteem implies that associations of positive traits with the self are stronger than associations of negative traits with the self. Based on the balance–congruity and the imbalance–dissonance principles, people will identify more strongly with groups that share self-associated positive traits. Correspondingly, the self-associated groups will be evaluated more positively than self-dissociated groups (i.e., in-group favoritism). This effect should be augmented the more positive people’s personal self-esteem is.

Several pieces of evidence support these propositions. In studies on the roots of intergroup bias, researchers often categorize participants into one of two different groups, based on an ostensible test on arbitrary personal characteristics that people share with a group of other people (e.g., “figure-oriented information processors” versus “ground-oriented processors”; Tajfel et al., 1971; Otten and Moskowitz, 2000). Immediately afterward, they assess attitudes toward both groups using either self-report questionnaires or reaction time procedures. The assessed explicit and implicit intergroup attitudes typically reflect a preference for the in-group over the out-group (e.g., Ashburn-Nardo et al., 2001). Even though different reasons have been suggested for how categorization contributes to preferences for in-groups (Hewstone et al., 2002), positive self-esteem is one of the established reasons (Cadinu and Rothbart, 1996; Gramzow and Gaertner, 2005; DiDonato et al., 2011; Roth and Steffens, 2014). In line with this account, research has demonstrated that individuals describe novel in-groups with traits that they also ascribe to themselves (Otten and Wentura, 2001; Otten, 2003). Research also has shown that people use self-ascribed traits to describe well-established in-groups such as gender groups (Otten and Epstude, 2006). Additionally, studies have shown that the more positive a person’s self-esteem, the more they prefer in-groups to out-groups (Aberson et al., 2000; also see Rudman et al., 2001). The evidence suggests that associating the self and social groups plays an important role in explicit (Gramzow and Gaertner, 2005) as well as implicit intergroup bias (Roth and Steffens, 2014). Additionally, these predictions have been supported using connectionist simulations (Vanhoomissen and Van Overwalle, 2010). Altogether, research has provided clear evidence that explicit and implicit preferences for self-associated groups (i.e., in-groups) over self-dissociated groups (out-groups) form in line with the balance–congruity principle and the imbalance–dissonance principle (Greenwald et al., 2002; Dunham, 2013; Cvencek et al., 2016).

### Predictions for How Changing Group Membership Affects Intergroup Attitudes

Within the present model, we suggest that changes in group membership modify the associative structure among the self, traits, and social groups in line with the balance–congruity principle and the imbalance dissonance principle. These principles suggest that if a new in-group is compatible with previous in-groups the network forms associations between the self and the new in-group that coexists with associations of the previous in-groups and the self. If, however, the new in-group is incompatible with previous in-groups that are already associated with the self, the network resists forming associations with the new in-group or it dissociates previous in-groups from the self in favor of associating the new in-group with the self (see Hypotheses 1–3). How will these processes affect intergroup attitudes?

The predictions of the model for intergroup attitudes are straightforward. Based on the premise that most people have positive self-esteem, people will associate more strongly social groups with the self that share positive traits than groups that are associated with negative traits. Based on the balance–congruity principle the groups that are simultaneously associated with traits that are shared with the self will be evaluated in line with the self and thus, more positively than social groups that are less strongly associated with the self. Thus, the model implies that intergroup bias among compatible in-groups declines whereas in the case of incompatible in-groups people will show intergroup bias in favor of the group that is more strongly associated with the self.

Hypothesis 4: All else being equal, attitudes will be more positive toward the in-group (previous in-group or new in-group) that people identify more strongly with compared to the in-group (previous in-group or new in-group) that people identify less strongly with.
Hypothesis 5: Intergroup bias between a previous in-group and a new in-group will be stronger to the extent that the two groups are incompatible.

Previous research shows some preliminary evidence in support of Hypothesis 4 (Clement and Krueger, 2002; Sassenberg and Wieber, 2005). In the model, we have specified two ends of a continuum of changes in group membership. First, we considered changes of group membership that result in dissociating the original in-group from the self while the original out-group becomes associated to the self. In the laboratory, these situations have been created by categorizing people into one of two unfamiliar social groups, for example, by the bogus psychological test mentioned above (Otten and Wentura, 1999). Afterward, these people were informed about a more precise test of membership that revealed their opposite group membership (Clement and Krueger, 2002). In one of our studies, we informed people about a mistaken first assignment because of computer malfunction (Roth, 2010). In these situations, the model predicts that intergroup bias will flip – because group membership is the only information about the social groups and consequently only the group that people share membership with is associated with the self. To our knowledge, only one published experiment has investigated effects of changing membership between in-group and out-group (Clement and Krueger, 2002). In this experiment, reassigning group membership changed which of the groups participants projected their own opinion on: Irrespective of prior group membership, participants projected to their new in-group (original out-group). Even though this experiment did not directly focus on intergroup attitudes, it provides preliminary evidence that cognitions about social groups switch when membership in unfamiliar social groups changes and the out-group is associated with the self instead of the previous in-group.

Outside the laboratory, however, situations in which people completely dissociate a previous in-group from the self could be rare. Instead, people may change group membership and associate a new in-group with their self but keep residual associations with the previous in-group due to its association with significant autobiographical memories. Thus, the new in-group is associated with the positive self in addition to the previous in-group. Consequently, in this case intergroup bias should be weaker because both groups are associated with the self. However, the imbalance–dissonance principle suggests that either the previous in-group or the new in-group is dissociated from the self the more incompatible the groups are with each other. Dissociating the previous in-group from the self while associating the new in-group with the self will result in preferences for the new in-group over the previous in-group; whereas dissociating the new in-group from the self, while associating the previous in-group with the self will result in preferences for the previous in-group. Likewise, the model’s balance–congruity principle suggests simultaneously strong associations of the self and the previous in-group as well as the new in-group when both groups are compatible which will result in a decline of intergroup bias.

We have elaborated on two ends of what we suggest to be a continuum of changes in group membership. In summary, the model suggests that the relative attitude toward a previous in-group and a new in-group changes depending on the (in)compatibility of the social groups. We believe that most kinds of changes of group membership affect the relative association of the self with the previous and new in-groups with effects on intergroup attitudes. Furthermore, with respect to groups that people never belonged to, we derive the following hypothesis.

Hypothesis 6: The group people were a member of and the group they become a member of will be evaluated more positively than social groups that people have not been a member of at any point in time.

## Resistant Versus Malleable Intergroup Attitudes

Research during the last decades has distinguished more automatic and less reflective attitudes (i.e., implicit attitudes) from less automatic and more reflective attitudes (i.e., explicit attitudes). Explicit attitudes are usually measured with self-report questionnaires. Answering questionnaires allows research participants to control and adjust their answers to what they consider an appropriate or correct attitude. Therefore, explicit attitudes are sensitive to social desirability and other controlled influences on attitudes. Implicit attitudes are measured indirectly, usually without the participant knowing the precise purpose of the task. Implicit attitudes are often inferred from reaction time differences in simple categorization tasks (e.g., Implicit Association Test, Greenwald et al., 1998; evaluative priming, Fazio, 1995). In general, implicit attitudes are considered to reflect more directly the underlying associative base of an attitude than explicit attitudes do (Fazio and Olson, 2003; Conrey et al., 2005; Gawronski and Sritharan, 2010). Distinguishing between implicit and explicit attitudes can help understanding of why people’s attitude toward a social group in one situation differs from their attitude toward the same group in a different situation.

In line with this approach, models of implicit and explicit attitudes share the assumption that implicit and explicit attitudes can differ. However, it is still debated why and when they differ. Early theory conceptualized implicit attitudes as unconscious, and therefore not available to introspection, which is suggested to be a precondition for explicit attitudes (Greenwald and Banaji, 1995). Other models propose two different systems that hardly communicate. Within these models, the implicit attitude is claimed to be overlearned and stored in a distinct system. It is considered to be difficult to change whereas the explicit attitude is easily updated in response to new information (e.g., Wilson et al., 2000; Rydell and McConnell, 2006). In contrast to these claims, evidence has accumulated that people are well aware of their implicit attitudes and that they are able to introspect about them (Hahn et al., 2014). Furthermore, implicit attitudes are less stable over time than explicit attitudes, which speaks against the hypothesis that implicit attitudes are in general overlearned and harder to change than explicit attitudes (Gawronski et al., 2017). Finally, research shows that implicit and explicit attitudes correlate depending on boundary conditions such as motivation and cognitive resources (Amodio et al., 2003; Hofmann et al., 2005). Some other models that coincide with these findings suggest two different systems or processes that do interact (e.g., Strack and Deutsch, 2004; Cunningham et al., 2007) and that operating conditions determine whether implicit and explicit attitudes diverge or converge (Olson and Fazio, 2009). Two of these models specifically address attitude change (Gawronski and Bodenhausen, 2006; Petty et al., 2007). Both models suggest that implicit attitudes more directly reflect the underlying associative structure than explicit attitudes. Furthermore, implicit attitudes serve as the basis for explicit attitudes, unless the information implied by the implicit attitude is rejected because of its inconsistency with other considered information. If information that is considered valid diverges from whichever associative structure is automatically activated, the implicit attitude reflects the automatic activated pattern whereas the explicit attitude is adjusted to the information considered to be valid (Gawronski and Bodenhausen, 2007). Most importantly, these models suggest that changes in the associative structure are reflected more directly in the implicit attitude whereas the explicit attitude additionally reflects information deemed to be valid.

Experimental evidence on the resistance versus malleability of implicit attitudes compared to explicit attitudes is ambiguous. Importantly, these divergent outcomes resulted from very different attitude manipulations (also see Rudman, 2004). Some studies provided information to participants that a recently established attitude was based on mistaken descriptive information (e.g., Gregg et al., 2006), whereas other studies used evaluative conditioning to change attitudes (e.g., Dijksterhuis, 2004). Whilst evaluative conditioning changed implicit attitudes but not explicit ones (e.g., Rydell et al., 2006; Grumm et al., 2009), verbal information changed explicit attitudes but not implicit ones (e.g., Gregg et al., 2006; Rydell et al., 2006). In sum, studies suggest that implicit and explicit attitude change can depend on the approach used to alter attitudes and on the processes involved in the respective approach (e.g., Mann and Ferguson, 2015; Wyer, 2016). In our reading, implicit attitudes change when the associative pattern changes, whereas corresponding changes in explicit attitudes will be moderated depending on whatever is considered valid (Gawronski and Bodenhausen, 2011) or appropriate (Olson and Fazio, 2009).

Will changing group membership and the resulting change in self-group associations affect explicit and implicit intergroup attitudes similarly? In line with established attitude models (Gawronski and Bodenhausen, 2006; Petty et al., 2007), we expect changes in the associative structure among the self, attributes, and the social groups to be reflected more directly in implicit intergroup attitudes. Thus, the effect of changes in group membership and its resulting effect on self-group associations in line with the balance–congruity and the imbalance–dissonance principles should more strongly be observed on implicit intergroup attitudes. These changes will not necessarily show in explicit intergroup attitudes. Explicit intergroup attitudes will depend on what information people consider most valid. Importantly, several sources influence what people consider valid information about a social group when they become a member themselves (e.g., Abrams and Hogg, 1988; Hogg and Abrams, 1993). These validation processes (Gawronski and Bodenhausen, 2011) can overwrite effects of associations between the self and the previous and the new in-group membership on their explicit intergroup attitudes.

Briefly, we suggest that associating the self to a previous out-group by changing group membership is a mechanism by which implicit intergroup attitudes can switch when the previous in-group is disconnected from the self. Implicit intergroup bias should decline when a new in-group is associated similarly strong with the self than the previous in-group. When groups differ on other dimensions in addition to the relative associations between the self and each of the groups, self-group associations will alter the implicit intergroup attitude. However, group stereotypes and any validating forces should influence explicit intergroup attitudes because explicit attitudes reflect the information that people consider most valid and appropriate (Gawronski and Bodenhausen, 2006). This leads us to our final hypothesis.

Hypothesis 7: Effects on intergroup attitudes as stated in Hypotheses 4 and 5 will be shown more clearly in measures that more proximally assess associative patterns underlying attitudes than on self-reported attitudes.

## Integration With Theories on Multiple Identities

The present model complements previous theories on identity change and multiple social identities. Previous models have suggested that identity integration depends on the compatibility or overlap of social groups (e.g., Benet-Martínez and Haritatos, 2005; Amiot et al., 2007) and that a non-overlap between social groups goes along with intrapsychic conflicts, discrimination and reduced well-being (Amiot et al., 2015). The present model suggests that these relationships can be traced back to the representation of the self and social groups in the mental knowledge structure. The model builds on cognitive consistency principles in the formation and change of mental associations among concepts. Research has demonstrated that cognitive dissonance is reflected in a tension and negative emotions (Levy et al., 2017) which is in line with the assumption that incompatibility of social identities goes along with negative outcomes. The present model specifies cognitive mechanisms that contribute to the developmental stages of identity integration described in the model of Amiot et al. (2007). It also accounts for the suggestion in work on bicultural identity integration by Benet-Martínez and Haritatos (2005) that the perceived distance between two cultures goes along with acculturation stress and identification with either one instead of both cultures (Benet-Martínez and Haritatos, 2005). Thus, the present model provides a general framework on the underlying cognitive mechanisms involved in integrating multiple social identities as specified by previous models.

Another prominent model that addresses the integration of multiple social groups into the self-concept is the identity complexity model by Roccas and Brewer (2002). These authors suggest that the less identities overlap (thus, the less compatible they are) the more complex will be the representation of in-groups in the mental knowledge structure and that this complexity is beneficial for people’s well-being and for intergroup relations. At first glance, this prediction seems to be at odds with the present model. The present model suggests that incompatibility between social groups results in discrepancies in the strength of identification with the groups: People will identify with one of the incompatible in-groups more strongly than with the other one. Furthermore, according to the present model, people should show preferences for the group that is more strongly associated with the self than with groups that are less strongly associated with the self. However, when people are members of different social groups that contradict each other in some characteristics, other characteristics may counteract dissociating any one of the social groups from the self. In order to restore cognitive consistency without dissociating any one of these in-groups from the self may result in a differentiation of social groups. For example, if a person identifies with women and similarly identifies with math majors, differentiating the concept women into math-affiliated women and math-opposing women can reduce dissonance between both in-groups (to apply this mechanism to stereotype threat theory, compare to Schmader et al., 2008). Thus, when incompatibility between social groups repeatedly leads to cognitive dissonance and disidentification with any one of the conflicted group memberships is not possible, these pressured concepts will tend to split into subconcepts (Greenwald et al., 2002). This dissonance resolving differentiation process may account for the premise of Roccas and Brewer’s (2002) identity complexity model. Thus, whereas the present model makes clear predictions about relative group identification with (in)compatible in-groups, incompatibility between in-groups may progressively be resolved by a process of differentiation.

Interestingly, Crisp and Turner (2011) have suggested that being confronted with multiple social identities results in flexible inhibition of stereotypes. These authors argued that the incompatibility of different in-groups’ stereotypes can be progressively resolved by repeated confrontation resulting in flexible inhibition of stereotypes. Although the authors reviewed convincing evidence on the perception of out-group members that disconfirm existing stereotypes, there seems to be only little evidence on whether the same processes hold true for conflicting in-groups. Therefore, it is an interesting question whether the perceived incompatibility of in-groups is resolved in the ways suggested by Crisp and Turner (2011).

## Implications for Reducing Intergroup Bias

Reducing intergroup bias is important, and policy makers undertake efforts toward this goal. Moreover, in contemporary societies changing group membership is common. The causes of intergroup bias (and changing group membership) are complex and we are looking at just one aspect of this. Hence, there will be many possible strategies for reducing bias, and we are not suggesting that our model provides a full solution. However, based on the model we address some points that could be helpful. First, in line with previous research, the present model claims that group identification is a driving force of intergroup bias (Sassenberg and Wieber, 2005). Second, the model provides a framework specifying when and how intergroup bias can alter, based on changes of group membership. We do not suggest that the relative strength of self-group associations is the only source of intergroup bias, but it can serve as a resource for altering explicit as well as implicit intergroup bias. Therefore, the model suggests that any intervention that helps perceiving similarities among several social groups and that fit with people’s self-concept can be effective in reducing intergroup bias between these groups. These predictions of the present model are in line with Gaertner and Dovidio’s (2012) common in-group identification model. These authors suggest that perceiving out-groups as part of a broader inclusive in-group reduces intergroup bias. Whereas their model focuses on the hierarchical integration of social identities into the self-concept with consequences for social groups that are included in an overarching category, the present model focusses on intergroup attitudes among groups that people are member of or have been member of at a certain point in time. Thus, both approaches complement each other.

An example of evidence in line with the present model is that exchange programs for students reduced intergroup bias by increasing the overlap between students enrolled in the program and the respective national out-group (Sassenberg and Matschke, 2010). From the perspective of the present model, exchange programs can be one way of highlighting commonalities between nationalities. Consequently, the foreign nationality and the original nationality can both become associated with the self-concept, resulting in a decline of intergroup bias (for similar findings, see extended contact effects, e.g., Wright et al., 1997). This is in line with cross-categorization models that suggest that double in-groups (e.g., a German US citizen) are evaluated more positively than cross-categories that include only one in-group and an out-group (e.g., Hewstone et al., 1993). When the person self-associates with Germans and United States citizens both social groups are associated with traits that are shared with the self. However, when only one of the groups is associated with the self and the other social group is incompatible with that in-group, the crossed category should be evaluated less in line with self-ascribed attributes.

The processes outlined in the present model mirror those described in models on the effect of perspective taking on out-group attitudes (e.g., Todd and Burgmer, 2013). In fact, it appears that the suggested mechanisms involved in changing group membership may also account for reduction of bias when people take the perspective of another person. Thus, the present model suggests that perspective taking will be more effective in improving out-group attitudes when the out-group is compatible instead of incompatible with relevant in-groups.

The present model contributes to understanding the formation and change of intergroup bias. It helps predicting when and how changes of group membership affect intergroup attitudes. This knowledge can inform the important societal issue of intergroup bias in a world where some group boundaries are becoming more malleable. The model suggests simple mechanisms, based on cognitive consistency principles, implying that new in-groups that are compatible with previous in-groups are associated with the self in line with previous identities and that associating social groups to this knowledge structure increases liking for these groups. Strengthening and reducing these connections can increase and decrease bias between social groups. Interventions that help highlighting commonalities instead of contradictions between social groups and thereby help the integration of new groups into the self-concept in line with previous identities may help changing intergroup bias. This has important implications for assimilation policies in multi-cultural countries. Policies should encourage compatibility between previous and new identities. This implies that people from different cultures need to go to the same schools, hold similar jobs, and live in similar places. It discourages any kind of segregation since this may increase incompatibility between previous and new group memberships. Since compatibility between social groups may be reached by assimilating a minority group membership to a majority group but also vice versa, it could be a promising strategy to make salient aspects of the majority’s preexisting identity that are consistent with including the minority in their group (also see Smeekes and Verkuyten, 2015). These strategies may help to reach an identity integration stage that implies high involvement in the culture of origin as well as the new cultural in-group (Berry, 1997) and represents the most adaptive of four different acculturation categories (Berry, 2005).

## Future Directions

The present model builds on cognitive consistency principles in social cognition to explain the integration of new identities into the self-concept with consequences for intergroup bias. Based on these established mechanisms, the present model suggests that group identification is represented in the mental knowledge structure by an associative link between the self and the in-group. The model suggests that changes in group membership affect the cognitive representation of the self and the in-group and out-group depending on group (in)compatibility that in turn affects intergroup attitudes. We stated hypotheses that we believe are the crucial or most interesting ones for the question how changes in group membership affect group identification and intergroup bias, but this is not an exhaustive list of hypothesizes that can be derived from the model. Systematic research is warranted to test the hypotheses derived from the model. In a first step, this could be done by using minimal groups to which people are assigned and reassigned (Clement and Krueger, 2002). Minimal groups have the advantage that people do not have previous stereotypes or experiences with these groups. This opens up the possibility to manipulate group stereotypes, norms, values, and goals to be compatible or incompatible between the social groups. Furthermore, additional variables such as group member’s experiences with these groups or duration of group membership can be manipulated. In order to address concerns with external validity of minimal group experiments, research should test the model’s prediction with existing groups and memberships. Thus, in a second step, the variables of interest could be measured or quasi-experimentally manipulated. Finally, longitudinal research on changes in group membership could show these change processes occurring naturally.

Notably, several motivational, situational, and interindividual differences may influence the strength of the mental association between the self and the original in-group as well as the self and the new in-group. In this paragraph, we mention some of the factors that could be addressed in future research. We believe that one crucial variable that influences the strength of the association between the self and a social group is the duration of the respective group membership. Whereas a continuous history of a person as member of a social group may intensify the association between the self and that group, a short-term membership without much meaning may work to the contrary. Short-term group membership may end up in a loose association between the self and the group that is more easily overwritten when membership changes; more permanent group membership may strengthen the connection (Sanchez et al., 2007). Autobiographical memory about the self as a member of a social group that goes along with an extensive duration of group membership may affect attitudes toward that group long after the person left that group. This should be particularly the case when the previous in-group shares stable characteristics with the self. For example, when people are forced to change their group membership and the new in-group is incompatible with the previous in-group people will show stronger associations with the previous in-group than with the new in-group (e.g., Jetten and Hutchison, 2011).

Furthermore, motivational processes may play a role in associating or dissociating a social group to/from the self. Research has shown that people identify with social groups to satisfy basic motives (Vignoles et al., 2006, 2008). Thus, people may more strongly associate social groups to their self-concept that fulfill their motives for self-esteem, distinctiveness, continuity, and meaning, whereas social groups that do not satisfy these motives may be associated less strongly to the self-concept. These motives may be reflected in variations of how much people voluntarily belong to a social group. Similarly, group membership based on shared opinion between the self and a social group may lead to strong identification with opinion-based groups with the result of strong intergroup bias toward the opposing group and consequences for collective action (Bliuc et al., 2007). In general, we predict that the more people want to belong to a social group the stronger they will identify with this group and thus, show a stronger association between the self and the social group. This has consequences for intergroup attitudes; the chosen in-group will be evaluated positively and because of its incompatibility with opponent groups, the latter may be devalued. It is another interesting question to test whether social groups that people disidentify with as measured with explicit measures (Becker and Tausch, 2014) can still be associated with the self-influencing intergroup attitudes (Kachanoff et al., 2016). The present model suggests that the more positive people’s self-esteem is the stronger their intergroup bias will be in favor of the group that is more strongly connected with the self. This process does not preclude additional motivational processes such as the deliberate selection of positive characteristics when describing the in-group compared to the out-group. Future research could test the relative contribution of these processes to intergroup bias. Finally, a central proposition of the model is that most people’s self-esteem is positive and that associating the self with a social group therefore contributes to a preference of the self-associated over a self-dissociated social group. However, some people’s self-esteem is negative; future research should test the generalizability of the predictions derived from the present model across various populations (e.g., people with affective disorders or those who belong to stigmatized groups).

## Conclusion

We presented a parsimonious model on how changes of group membership are reflected in cognitive representations of the self, traits, and the group’s (in)compatibility that in turn can alter implicit and also explicit intergroup bias. However, the influence on explicit intergroup bias is also affected by what a person considers an appropriate attitude in the respective situation. Our cognitive consistency based recategorization model suggests that any group that is associated with the self-profits from self-associated positive valence and is thus represented more positively than groups that are linked less strongly or are dissociated from the self. Based on established cognitive consistency principles and on previous research on categorization-induced intergroup bias, self-anchoring, and dual process perspectives on attitudes, the present model suggests that not only explicit but also implicit intergroup bias is flexible, and that it depends on associating social groups to the individual’s self-concept. The model suggests that self-association is a process that in addition to established mechanisms like evaluative conditioning can effectively change the associative structure underlying the self-concept and social groups. Altogether, changing group membership can be an effective strategy in altering reflected as well as more automatic forms of intergroup bias – at least when boundaries between groups are permeable. Given that contemporary societies are characterized by frequent changes of group memberships, be it related to jobs, professions, cities, or countries, this article aims to shift from a relatively ‘static’ consideration of social group memberships in the here and now to a more ‘flexible’ view of group memberships as changing through space and time. In modern society, group memberships change, and so do social identities. Given that social identities are important aspects of the individual’s self-concept, identity change is complex and important. The suggested model provides a framework from which innovative testable predictions can be derived and tested that in turn help to explain and predict societally relevant contemporary issues of modern life.

## Author Contributions

JR drafted the manuscript. MS and VV provided critical revision of the manuscript. All authors approved the final manuscript.

## Conflict of Interest Statement

The authors declare that the research was conducted in the absence of any commercial or financial relationships that could be construed as a potential conflict of interest.

## References

[B1] Aberson C. L. Healy M. Romero V. (2000). Ingroup bias and self-esteem: a meta-analysis. *Pers. Soc. Psychol. Rev.* 4 157–173. 10.1207/S15327957PSPR0402_04

[B2] Abrams D. Hogg M. A. (1988). Comments on the motivational status of self-esteem in social identity and intergroup discrimination. *Eur. J. Soc. Psychol.* 18 317–334. 10.1002/ejsp.2420180403

[B3] Alicke M. D. Govorun O. (2005). “The better-than-average effect,” in *The Self in Social Judgment*, eds Alicke M. D. Dunning D. A. Krueger J. I. (New York, NY: Taylor and Francis), 85–106.

[B4] Allport G. W. (1954). *The Nature of Prejudice.* Reading, MA: Addison-Wesley.

[B5] Amiot C. E. De La Sablonniere R. Smith L. G. Smith J. R. (2015). Capturing changes in social identities over time and how they become part of the self-concept. *Soc. Pers. Psychol. Compass* 9 171–187. 10.1111/spc3.12169

[B6] Amiot C. E. De La Sablonniere R. Terry D. J. Smith J. R. (2007). Integration of social identities in the self: toward a cognitive-developmental model. *Pers. Soc. Psychol. Rev.* 11 364–388. 10.1177/1088868307304091 18453468

[B7] Amodio D. M. Harmon-Jones E. Devine P. G. (2003). Individual differences in the activation and control of affective race bias as assessed by startle eyeblink response and self-report. *J. Pers. Soc. Psychol.* 84 738–753. 10.1037/0022-3514.84.4.738 12703646

[B8] Ashburn-Nardo L. Voils C. I. Monteith M. J. (2001). Implicit associations as the seeds of intergroup bias: how easily do they take root? *J. Pers. Soc. Psychol.* 81 789–799. 10.1037//0022-3514.81.5.789 11708557

[B9] Becker J. C. Tausch N. (2014). When group memberships are negative: the concept, measurement, and behavioral implications of psychological disidentification. *Self Identity* 13 294–321. 10.1080/15298868.2013.819991

[B10] Benet-Martínez V. Haritatos J. (2005). Bicultural identity integration (BII): components and psychosocial antecedents. *J. Pers.* 73 1015–1050. 10.1111/j.1467-6494.2005.00337.x 15958143

[B11] Berry J. W. (1997). Immigration, acculturation, and adaptation. *Appl. Psychol.* 46 5–34. 10.1111/j.1464-0597.1997.tb01087.x

[B12] Berry J. W. (2005). Acculturation: living successfully in two cultures. *Int. J. Intercult. Relat.* 29 697–712. 10.1016/j.ijintrel.2005.07.013

[B13] Bliuc A. M. McGarty C. Reynolds K. Muntele D. (2007). Opinion-based group membership as a predictor of commitment to political action. *Eur. J. Soc. Psychol.* 37 19–32. 10.1002/ejsp.334

[B14] Bosson J. K. Swann W. B. Pennebaker J. W. (2000). Stalking the perfect measure of implicit self-esteem: the blind men and the elephant revisited? *J. Pers. Soc. Psychol.* 79 631–643. 10.1037//0022-3514.79.4.631 11045743

[B15] Brewer M. B. (1979). In-group bias in the minimal intergroup situation: a cognitive-motivational analysis. *Psychol. Bull.* 86 307–324. 10.1037/0033-2909.86.2.307

[B16] Brewer M. B. (2000). “Reducing prejudice through cross-categorization: effects of multiple social identities,” in *Claremont Symposium on Applied Social Psychology: Reducing Prejudice and Discrimination*, ed. Oskamp S. (Thousand Oaks, CA: Sage), 165–183.

[B17] Brewer M. B. Weber J. G. (1994). Self-evaluation effects of interpersonal versus intergroup social comparison. *J. Pers. Soc. Psychol.* 66 268–275. 10.1037/0022-3514.66.2.268 8195985

[B18] Brown J. D. (2012). Understanding the better than average effect: motives (Still) matter. *Pers. Soc. Psychol. Bull.* 38 209–219. 10.1177/0146167211432763 22205623

[B19] Cadinu M. R. Rothbart M. (1996). Self-anchoring and differentiation processes in the minimal group setting. *J. Pers. Soc. Psychol.* 70 661–677. 10.1037/0022-3514.70.4.661 8636892

[B20] Clement R. W. Krueger J. (2002). Social categorization moderates social projection. *J. Exp. Soc. Psychol.* 38 219–231. 10.1006/jesp.2001.1503 27538410

[B21] Conrey F. R. Sherman J. W. Gawronski B. Hugenberg K. Groom C. J. (2005). Separating multiple processes in implicit social cognition: the quad model of implicit task performance. *J. Pers. Soc. Psychol.* 89 469–487. 10.1037/0022-3514.89.4.469 16287412

[B22] Crisp R. J. Turner R. N. (2011). Cognitive adaptation to the experience of social and cultural diversity. *Psychol. Bull.* 137 242–266. 10.1037/a0021840 21142349

[B23] Cunningham W. A. Zelazo P. D. Packer D. J. Van Bavel J. J. (2007). The iterative reprocessing model: a multilevel framework for attitudes and evaluation. *Soc. Cogn.* 25 736–760. 10.1521/soco.2007.25.5.736 25168638

[B24] Cvencek D. Greenwald A. G. Meltzoff A. N. (2012). “Balanced identity theory: review of evidence for implicit consistency in social cognition,” in *From Cognitive Consistency: A Fundamental Principle in Social Cognition*, eds Gawronski B. Strack F. (New York, NY: The Guilford Press), 157–177.

[B25] Cvencek D. Greenwald A. G. Meltzoff A. N. (2016). Implicit measures for preschool children confirm self-esteem’s role in maintaining a balanced identity. *J. Exp. Soc. Psychol.* 62 50–57. 10.1016/j.jesp.2015.09.015

[B26] DiDonato T. E. Ullrich J. Krueger J. I. (2011). Social perception as induction and inference: an integrative model of intergroup differentiation, ingroup favoritism, and differential accuracy. *J. Pers. Soc. Psychol.* 100 66–85. 10.1037/a0021051 20939650

[B27] Dijksterhuis A. (2004). I like myself but I don’t know why: enhancing implicit self-esteem by subliminal evaluative conditioning. *J. Pers. Soc. Psychol.* 86 345–355. 10.1037/0022-3514.86.2.345 14769089

[B28] Dovidio J. F. Gaertner S. L. Hodson G. Riek B. M. Johnson K. M. Houlette M. (2006). “Recategorization and crossed categorization: the implications of group salience and representations for reducing bias,” in *Multiple Social Categorization: Processes, Models and Applications*, eds Crisp R. J. Hewstone M. Crisp R. J. Hewstone M. (New York, NY: Psychology Press), 65–89.

[B29] Dunham Y. (2013). Balanced identity in the minimal groups paradigm. *PLoS One* 8:e84205. 10.1371/journal.pone.0084205 24391912 PMC3877254

[B30] Ensari N. Miller N. (2001). Decategorization and the reduction of bias in the cross categorization paradigm. *Eur. J. Soc. Psychol.* 31 193–216. 10.1002/ejsp.42

[B31] Falk C. F. Heine S. J. (2015). What is implicit self-esteem, and does it vary across cultures? *Pers. Soc. Psychol. Rev.* 19 177–198. 10.1177/1088868314544693 25063044

[B32] Fazio R. H. (1995). “Attitudes as object-evaluation associations: determinants, consequences, and correlates of attitude accessibility,” in *Attitude Strength: Antecedents and Consequences*, eds Petty R. E. Krosnick J. A. (Hillsdale, NJ: Erlbaum), 247–282.

[B33] Fazio R. H. Olson M. A. (2003). Implicit measures in social cognition research: their meaning and uses. *Annu. Rev. Psychol.* 54 297–327. 10.1146/annurev.psych.54.101601.145225 12172003

[B34] Festinger L. (1957). *A Theory of Cognitive Dissonance.* Palo Alto, CA: Stanford University Press.

[B35] Franck E. Raedt R. D. de Houwer J. D. (2007). Implicit but not explicit self-esteem predicts future depressive symptomatology. *Behav. Res. Ther.* 45 2448–2455. 10.1016/j.brat.2007.01.008 17343822

[B36] Gaertner S. L. Dovidio J. F. (2005). “Categorization, recategorization, and intergroup bias,” in *Reflecting on the Nature of Prejudice: Fifty Years after Allport*, eds Dovidio J. F. Glick P. Rudman L. A. (Oxford: Blackwell Publishing), 71–88.

[B37] Gaertner S. L. Dovidio J. F. (2012). “The common ingroup identity model,” in *Handbook of Theories of Social Psychology* Vol. 2 eds Van Lange P. A. M. Kruglanski A. W. Higgins E. T. (Thousand Oaks, CA: Sage Publications Ltd), 439–457. 10.4135/9781446249222.n48

[B38] Gaertner S. L. Dovidio J. F. Banker B. S. Houlette M. Johnson K. M. McGlynn E. A. (2000). Reducing intergroup conflict: From superordinate goals to decategorization, recategorization, and mutual differentiation. *Group Dyn.* 4 98–114. 10.1037/1089-2699.4.1.98

[B39] Gawronski B. Bodenhausen G. V. (2006). Associative and propositional processes in evaluation: an integrative review of implicit and explicit attitude change. *Psychol. Bull.* 132 692–731. 10.1037/0033-2909.132.5.692 16910748

[B40] Gawronski B. Bodenhausen G. V. (2007). Unraveling the processes underlying evaluation: attitudes from the perspective of the APE model. *Soc. Cogn.* 25 687–717. 10.1521/soco.2007.25.5.687

[B41] Gawronski B. Bodenhausen G. V. (2011). 2 The associative-propositional evaluation model: theory, evidence, and open questions. *Adv. Exp. Soc. Psychol.* 44 59–127. 10.1016/B978-0-12-385522-0.00002-0

[B42] Gawronski B. Morrison M. Phills C. E. Galdi S. (2017). Temporal stability of implicit and explicit measures. *Pers. Soc. Psychol. Bull.* 43 300–312. 10.1177/0146167216684131 28903689

[B43] Gawronski B. Sritharan R. (2010). “Formation, change, and contextualization of mental associations: determinants and principles of variations in implicit measures,” in *Handbook of Implicit Social Cognition: Measurement, Theory, and Applications*, eds Gawronski B. Payne B. K. (New York, NY: Guilford Press), 216–240.

[B44] Gramzow R. H. Gaertner L. (2005). Self-esteem and favoritism toward novel in-groups: the self as an evaluative base. *J. Pers. Soc. Psychol.* 88 801–815. 10.1037/0022-3514.88.5.801 15898876

[B45] Greenwald A. G. Banaji M. R. (1995). Implicit social cognition: attitudes, self-esteem, and stereotypes. *Psychol. Rev.* 102 4–27. 10.1037/0033-295x.102.1.4 7878162

[B46] Greenwald A. G. Banaji M. R. Rudman L. A. Farnham S. D. Nosek B. A. Mellott D. S. (2002). A unified theory of implicit attitudes, stereotypes, self-esteem, and self-concept. *Psychol. Rev.* 109 3–25. 10.1037//0033-295X.109.1.3 11863040

[B47] Greenwald A. G. Farnham S. D. (2000). Using the implicit association test to measure self-esteem and self-concept. *J. Pers. Soc. Psychol.* 79 1022–1038. 10.1037/0022-3514.79.6.1022 11138752

[B48] Greenwald A. G. Mcghee D. E. Schwartz J. L. K. (1998). Measuring individual differences in implicit cognition: the implicit association test. *J. Pers. Soc. Psychol.* 74 1464–1480. 10.1037/0022-3514.74.6.1464 9654756

[B49] Gregg A. P. Seibt B. Banaji M. R. (2006). Easier done than undone: asymmetry in the malleability of automatic preferences. *J. Pers. Soc. Psychol.* 90 1–20. 10.1037/0022-3514.90.1.1 16448307

[B50] Grumm M. Nestler S. Von Collani G. (2009). Changing explicit and implicit attitudes: the case of self-esteem. *J. Exp. Soc. Psychol.* 45 327–335. 10.1016/j.jesp.2008.10.006

[B51] Hahn A. Judd C. M. Hirsh H. K. Blair I. V. (2014). Awareness of implicit attitudes. *J. Exp. Psychol.* 143 1369–1392. 10.1037/a0035028 24294868 PMC4038711

[B52] Hebb D. O. (1949). *Organization of Behavior.* New York, NY: Wiley.

[B53] Heider F. (1958). *The Psychology of Interpersonal Relations.* New York, NY: Wiley 10.1037/10628-000

[B54] Hewstone M. Islam M. R. Judd C. M. (1993). Models of crossed categorization and intergroup relations. *J. Pers. Soc. Psychol.* 64 779–793. 10.1037/0022-3514.64.5.779 8505707

[B55] Hewstone M. Rubin M. Willis H. (2002). Intergroup bias. *Annu. Rev. Psychol.* 53 575–604. 10.1146/annurev.psych.53.100901.135109 11752497

[B56] Hofmann W. Gawronski B. Gschwendner T. Le H. Schmitt M. (2005). A meta-analysis on the correlation between the implicit association test and explicit self-report measures. *Pers. Soc. Psychol. Bull.* 31 1369–1385. 10.1177/0146167205275613 16143669

[B57] Hogg M. A. Abrams D. (1993). “Towards a single-process uncertainty-reduction model of social motivation in groups,” in *Group Motivation: Social Psychological Perspectives*, eds Hogg M. A. Abrams D. (Hertfordshire: Harvester Wheatsheaf), 173–190.

[B58] Huynh Q.-L. Nguyen A.-M. T. D. Benet-Martínez V. (2011). “Bicultural identity integration,” in *Handbook of Identity Theory and Research*, eds Schwartz S. J. Luyckx K. Vignoles V. L. (New York, NY: Springer Science + Business Media), 827–843. 10.1007/978-1-4419-7988-9_35

[B59] International Organization for Migration (2018). *World Migration Report 2018.* Geneva: International Organization for Migration.

[B60] Jetten J. Hutchison P. (2011). When groups have a lot to lose: historical continuity enhances resistance to a merger. *Eur. J. Soc. Psychol.* 41 335–343. 10.1002/ejsp.779

[B61] Jones J. M. Jetten J. (2011). Recovering from strain and enduring pain: multiple group memberships promote resilience in the face of physical challenges. *Soc. Psychol. Pers. Sci.* 2 239–244. 10.1177/1948550610386806

[B62] Kachanoff F. J. Ysseldyk R. Taylor D. M. Sablonnière R. Crush J. (2016). The good, the bad and the central of group identification: evidence of a u-shaped quadratic relation between in-group affect and identity centrality. *Eur. J. Soc. Psychol.* 46 563–580. 10.1002/ejsp.2199

[B63] Levy N. Harmon-Jones C. Harmon-Jones E. (2017). Dissonance and discomfort: does a simple cognitive inconsistency evoke a negative affective state? *Motiv. Sci.* (in press). 10.1037/mot0000079

[B64] Mann T. C. Ferguson M. J. (2015). Can we undo our first impressions? The role of reinterpretation in reversing implicit evaluations. *J. Pers. Soc. Psychol.* 108 823–849. 10.1037/pspa0000021 25798625 PMC4437854

[B65] Olson M. A. Fazio R. H. (2009). “Implicit and explicit measures of attitudes: The perspective of the MODE model,” in *Attitudes: Insights from the New Implicit Measures*, eds Petty R. E. Fazio R. H. Briñol P. (New York, NY: Psychology Press), 19–63.

[B66] Olson M. A. Fazio R. H. Hermann A. D. (2007). Reporting tendencies underlie discrepancies between implicit and explicit measures of self-esteem. *Psychol. Sci.* 18 287–291. 10.1111/j.1467-9280.2007.01890.x 17470249

[B67] Osgood C. E. Tannenbaum P. H. (1955). The principle of congruity in the prediction of attitude change. *Psychol. Rev.* 62 42–55. 10.1037/h0048153 14357526

[B68] Otten S. (2003). “Me and us” or “us and them”? The self as a heuristic for defining minimal ingroups. *Eur. Rev. Soc. Psychol.* 13 1–33. 10.1080/10463280240000028

[B69] Otten S. Epstude K. (2006). Overlapping mental representations of self, ingroup, and outgroup: unraveling self-stereotyping and self-anchoring. *Pers. Soc. Psychol. Bull.* 32 957–969. 10.1177/0146167206287254 16738028

[B70] Otten S. Moskowitz G. B. (2000). Evidence for implicit evaluative in-group bias: affect-biased spontaneous trait inference in a minimal group paradigm. *J. Exp. Soc. Psychol.* 36 77–89. 10.1006/jesp.1999.1399

[B71] Otten S. Wentura D. (1999). About the impact of automaticity in the minimal group paradigm: evidence from affective priming tasks. *Eur. J. Soc. Psychol.* 29 1049–1071. 10.1002/(SICI)1099-0992(199912)29:8<1049::AID-EJSP985>3.0.CO;2-Q

[B72] Otten S. Wentura D. (2001). Self-anchoring and in-group favoritism: an individual profiles analysis. *J. Exp. Soc. Psychol.* 37 525–532. 10.1006/jesp.2001.1479

[B73] Petty R. E. Briñol P. Demarree K. G. (2007). The meta-cognitive model (MCM) of attitudes: implications for attitude measurement, change, and strength. *Soc. Cogn.* 25 657–686. 10.1521/soco.2007.25.5.657

[B74] Roccas S. Brewer M. (2002). Social identity complexity. *Pers. Soc. Psychol. Rev.* 6 88–106. 10.1207/S15327957PSPR0602_01

[B75] Roth J. (2010). *Easily Liked and Disliked? Formation and Change of Explicit and Implicit Attitudes Towards Ones’s Group.* Ph.D. thesis, Friedrich-Schiller-Universität Jena, Jena.

[B76] Roth J. Steffens M. C. (2014). When I becomes we: associative self-anchoring drives implicit intergroup bias in minimal groups. *Soc. Psychol.* 45 253–264. 10.1027/1864-9335/a000169

[B77] Roth J. Steffens M. C. Morina N. Stangier U. (2012). Changed for the worse: subjective change in implicit and explicit self-esteem in individuals with current, past, and no posttraumatic stress disorder. *Psychother. Psychosom.* 81 64–66. 10.1159/000329993 22123458

[B78] Rudman L. A. (2004). Sources of implicit attitudes. *Curr. Dir. Psychol. Sci.* 13 79–82. 10.1111/j.0963-7214.2004.00279.x

[B79] Rudman L. A. Greenwald A. G. Mcghee D. E. (2001). Implicit self-concept and evaluative implicit gender stereotypes: self and ingroup share desirable traits. *Pers. Soc. Psychol. Bull.* 27 1164–1178. 10.1177/0146167201279009

[B80] Rydell R. J. McConnell A. R. (2006). Understanding implicit and explicit attitude change: a systems of reasoning analysis. *J. Pers. Soc. Psychol.* 91 995–1008. 10.1037/0022-3514.91.6.995 17144760

[B81] Rydell R. J. McConnell A. R. Mackie D. M. Strain L. M. (2006). Of two minds forming and changing valence-inconsistent implicit and explicit attitudes. *Psychol. Sci.* 17 954–958. 10.1111/j.1467-9280.2006.01811.x 17176426

[B82] Sanchez A. K. Zogmaister C. Arcuri L. (2007). When “they” becomes “we”: multiple contrasting identities in mixed status groups. *Self Identity* 6 154–172. 10.1080/15298860601128255

[B83] Sassenberg K. Matschke C. (2010). The impact of exchange programs on the integration of the hostgroup into the self-concept. *Eur. J. Soc. Psychol.* 40 148–159. 10.1002/ejsp.621

[B84] Sassenberg K. Wieber F. (2005). Don’t ignore the other half: the impact of ingroup identification on implicit measures of prejudice. *Eur. J. Soc. Psychol.* 35 621–632. 10.1016/j.jesp.2004.10.002

[B85] Schmader T. Johns M. Forbes C. (2008). An integrated process model of stereotype threat effects on performance. *Psychol. Rev.* 115 336–356. 10.1037/0033-295X.115.2.336 18426293 PMC2570773

[B86] Smeekes A. Verkuyten M. (2015). The presence of the past: identity continuity and group dynamics. *Eur. Rev. Soc. Psychol.* 26 162–202. 10.1080/10463283.2015.1112653

[B87] Strack F. Deutsch R. (2004). Reflective and impulsive determinants of social behavior. *Pers. Soc. Psychol. Rev.* 8 220–247. 10.1207/s15327957pspr0803_1 15454347

[B88] Tajfel H. (1974). Social identity and intergroup behaviour. *Soc. Sci. Informat.* 13 65–93. 10.1177/053901847401300204

[B89] Tajfel H. Billig M. G. Bundy R. P. Flament C. (1971). Social categorization and intergroup behaviour. *Eur. J. Soc. Psychol.* 1 149–178. 10.1002/ejsp.2420010202

[B90] Todd A. R. Burgmer P. (2013). Perspective taking and automatic intergroup evaluation change: testing an associative self-anchoring account. *J. Pers. Soc. Psychol.* 104 786–802. 10.1037/a0031999 23527849

[B91] Turner J. C. Hogg M. A. Oakes P. J. Reicher S. D. Wetherell M. S. (1987). *Rediscovering the Social Group: A Self-Categorization Theory.* Cambridge, MA: Basil Blackwell.

[B92] van Veelen R. Otten S. Cadinu M. Hansen N. (2015). An integrative model of social identification self-stereotyping and self-anchoring as two cognitive pathways. *Pers. Soc. Psychol. Rev.* 20 3–26. 10.1177/1088868315576642 25800408

[B93] Vanhoomissen T. Van Overwalle F. (2010). Me or not me as source of ingroup favoritism and outgroup derogation: a connectionist perspective. *Soc. Cogn.* 28 84–109. 10.1521/soco.2010.28.1.84

[B94] Vignoles V. L. Manzi C. Regalia C. Jemmolo S. Scabini E. (2008). Identity motives underlying desired and feared possible future selves. *J. Pers.* 76 1165–1200. 10.1111/j.1467-6494.2008.00518.x 18665893

[B95] Vignoles V. L. Regalia C. Manzi C. Golledge J. Scabini E. (2006). Beyond self-esteem: influence of multiple motives on identity construction. *J. Pers. Soc. Psychol.* 90 308–333. 10.1037/0022-3514.90.2.308 16536653

[B96] Wilson T. D. Lindsey S. Schooler T. Y. (2000). A model of dual attitudes. *Psychol. Rev.* 107 101–126. 10.1037/0033-295X.107.1.101 10687404

[B97] Wright S. C. Aron A. Mclaughlin-Volpe T. Ropp S. A. (1997). The extended contact effect: knowledge of cross-group friendships and prejudice. *J. Pers. Soc. Psychol.* 73 73–90. 10.1037/0022-3514.73.1.73

[B98] Wyer N. A. (2016). Easier done than undone… by some of the people, some of the time: the role of elaboration in explicit and implicit group preferences. *J. Exp. Soc. Psychol.* 63 77–85. 10.1016/j.jesp.2015.12.006 16448307

[B99] Yamaguchi S. Greenwald A. G. Banaji M. R. Murakami F. Chen D. Shiomura K. (2007). Apparent universality of positive implicit self-esteem. *Psychol. Sci.* 18 498–500. 10.1111/j.1467-9280.2007.01928.x 17576261

